# Ginsenoside Rb1 attenuates age-associated vascular impairment by modulating the Gas6 pathway

**DOI:** 10.1080/13880209.2021.1986076

**Published:** 2021-10-09

**Authors:** Shiye Ke, Lin Wu, Min Wang, Dinghui Liu, Guangyao Shi, Jieming Zhu, Xiaoxian Qian

**Affiliations:** a Department of Cardiology, The Third Affiliated Hospital of Sun Yat-Sen University, Guangzhou, China; b Department of Cardiology, The Eighth Affiliated Hospital of Sun Yat-Sen University, Shenzhen, China; c Institute of Integrated Traditional Chinese and Western Medicine, Sun Yat-Sen University, Guangzhou, China

**Keywords:** Ageing, vascular calcification, fibrosis, growth arrest-specific protein 6 (Gas6)/Axl pathway

## Abstract

**Context:**

Ginsenoside Rb1 (Rb1) exerts many beneficial effects and protects against cardiovascular disease.

**Objective:**

To investigate whether Rb1 could attenuate age-related vascular impairment and identify the mechanism.

**Materials and methods:**

Female C57BL/6J mice aged 2 and 18 months, randomly assigned to Young, Young + 20 mg/kg Rb1, Old + vehicle, Old + 10 mg/kg Rb1 and Old + 20 mg/kg Rb1 groups, were daily intraperitoneal injected with vehicle or Rb1 for 3 months. The thoracic aorta segments were used to inspect the endothelium-dependent vasorelaxation. Left thoracic aorta tissues were collected for histological or molecular expression analyses, including ageing-related proteins, markers relevant to calcification and fibrosis, and expression of Gas6/Axl.

**Results:**

We found that in Old + vehicle group, the expression of senescence proteins and cellular adhesion molecules were significantly increased, with worse endothelium-dependent thoracic aorta relaxation (58.35% ± 2.50%) than in Young group (88.84% ± 1.20%). However, Rb1 treatment significantly decreased the expression levels of these proteins and preserved endothelium-dependent relaxation in aged mice. Moreover, Rb1 treatment also reduced calcium deposition, collagen deposition, and the protein expression levels of collagen I and collagen III in aged mice. Furthermore, we found that the downregulation of Gas6 protein expression by 41.72% and mRNA expression by 52.73% in aged mice compared with young mice was abrogated by Rb1 treatment. But there was no significant difference on Axl expression among the groups.

**Conclusions:**

Our study confirms that Rb1 could ameliorate vascular injury, suggesting that Rb1 might be a potential anti-ageing related vascular impairment agent.

## Introduction

Over the next 30 years in China, there is an estimated increase from 12.6% to 26% of adults aged 65 and above (Zhou and Walker 2021). Although the human lifespan is increasing, age-related diseases continue to be a topic of concern. Cardiovascular diseases are the most common diseases that affect elderly individuals. Vascular ageing accompanied by vascular structural and functional changes is an important independent risk factor for cardiovascular diseases (Lakatta and Levy 2003). Epidemiological surveys have shown that 82% of patients who die of cardiovascular diseases are 65 years old or older (Moslehi et al. 2012). Therefore, exploring the mechanism of age-associated vascular impairment and finding agents that can protect vascular function against age-related decline might play important roles in reducing the incidence and prevalence of age-associated cardiovascular disorders in elderly individuals.

Arterial ageing has been implicated in the pathogenesis of vascular dysfunction and various cardiovascular diseases and is a hallmark of ageing (Minamino and Komuro 2007). Vascular alterations resulting from ageing vary and include endothelial dysfunction, hypertrophy of vascular smooth muscle cells (VSMCs), arterial dilatation, reorganisation of the extracellular matrix (ECM), vascular calcification, and an increase in the collagen-to-elastin ratio with fragmentation and vascular stiffing (Tolle et al. 2015). Brandes et al. (2005) reported that age-related endothelial dysfunction was commonly characterized by an imbalance in endothelium-derived relaxation and contractile factors. Further study by Herrera et al. (2010) also showed that endothelial dysfunction was closely associated with a progressive decline in endothelium-dependent vasodilatation. However, it is still unclear how ageing affects vasodilatation or triggers imbalances in related factors and ultimately leads to vascular dysfunction.

Acosta et al. (2008) showed that vascular cells undergoing senescence could produce adhesion molecules, including plasminogen activator inhibitor 1 (PAI-1), vascular cell adhesion protein 1 (VCAM-1) and intercellular cell adhesion molecule-1 (ICAM-1). All these adhesion molecules were thought to play important roles in monocyte recruitment to atherosclerotic sites and then initiate vascular remodelling, ultimately resulting in a reduction in compliance and an increase in stiffness (Acosta et al. 2008; Kovacic et al. 2011). Vascular stiffness is the most important manifestation of vascular ageing. It is attributed to ECM remodelling and contributes to a decline in vasodilatation. Previous studies have demonstrated that vascular stiffness and VSMC senescence are attributed to medial calcification and collagen deposition (Mauriello et al. 1992; McClelland et al. 2006; Nakano-Kurimoto et al. 2009; Wendorff et al. 2015; Kim et al. 2018). Therefore, vasodilatation dysfunction might result from calcification and fibrosis. Reducing calcium or collagen deposition may delay ageing-related arterial impairment.

Ginsenoside Rb1 (Rb1) is one of the active components found in ginseng. Research has reported that Rb1 exerts many beneficial antisenescence and anti-apoptotic effects, particularly in protecting the myocardium and endothelium (Zheng et al. 2017; Zhou et al. 2017; Zheng et al. 2020). Recent studies have demonstrated that Rb1 can reduce chronic kidney disease-associated vascular calcification and type I collagen expression in rats (Zhou et al. 2019b). Findings have also suggested that Rb1 reduces type I collagen expression (Kwok et al. 2012). However, the effects of Rb1 on age-associated vascular impairment have not been investigated.

It has been reported that Rb1 inhibits vascular calcification through growth arrest-specific gene 6 (Gas6) transactivation *in vitro* (Nanao-Hamai et al. 2019). Gas6, a member of the vitamin K-dependent protein family, is implicated in the regulation of multiple cellular functions after binding to its receptor Axl, a membrane receptor tyrosine kinase (Nakano et al. 1997; Fridell et al. 1998; Yanagita et al. 2001). Some studies have indicated that the Gas6/Axl pathway plays a pivotal role in vascular biology and diseases such as vascular calcification vascular remodelling and atherosclerosis (Korshunov et al. 2006; Hurtado et al. 2010; Son et al. 2010). We hypothesize that Rb1 can attenuate age-related vascular impairment by suppressing calcification and fibrosis, which is potentially associated with regulation of the Gas6/Axl pathway.

## Materials and methods

### Experimental regents

Rb1 (purity greater than 98%) was obtained from Victory (Sichuan, China). Antibodies against p21^Cip1^ (ab188224), p16^INK4a^ (ab51243), ICAM-1 (ab179707), VCAM-1 (ab134047), PAI-1 (ab222754) and GAPDH (ab181603) were purchased from Abcam (MA, USA). Antibodies against collagen I (14695), collagen III (22734) and Axl (13196) were purchased from Proteintech Group (MA, USA). Antibodies against Gas6 (A8545) and p16^INK4a^ (A0262) were purchased from ABclonal Technology (Hubei, China). Modified Krebs-Henseleit (K-H) buffer, phenylephrine (a potent vasoconstrictor) and acetylcholine (Ach, an endothelial-dependent NO donor) were purchased from Sigma-Aldrich (MO, USA). All other reagents used were of analytical grade.

### Animals and management

This study was approved by the Institutional Animal Care and Use Committee (IACUC) of Sun Yat-Sen University. Most longevity studies have shown effects on females (Hsu et al. 2018; Qin et al. 2018); hence, we performed our long-term study using female mice. Female C57BL/6J mice aged 2 and 18 months old were obtained from the Centre of Experimental Animals, Sun Yat-Sen University. The mice were fed a normal diet and housed under controlled conditions (24 ± 2 °C and 50 ± 5% humidity) with 12 h light/dark photoperiods under specific pathogen-free conditions. 40 young female C57BL/6J mice at 2 months of age were randomly assigned to the Young and Young + 20 mg/kg Rb1 (Young + Rb1-20) groups (*n* = 20 each). These mice were treated with a daily intraperitoneal injection of vehicle (sterile saline solution) or 20 mg/kg Rb1 dissolved in vehicle for 3 months before euthanasia by CO_2_ asphyxiation at 5 months of age. 60 aged female C57BL/6J mice at 18 months of age were randomly assigned to the Old + vehicle, Old + 10 mg/kg Rb1 (Old + Rb1-10) and Old + 20 mg/kg Rb1 (Old + Rb1-20) groups (*n* = 20 each). The mice that survived until the end of this experiment received daily intraperitoneal injections of vehicle alone or Rb1 (10 or 20 mg/kg) for 3 months, after which the mice were euthanized at 21 months of age. Experiments were repeated at least three times with 4-5 mice per group.

### Determination of vasorelaxation in thoracic aortic rings

After the animals were euthanized, thoracic aortic rings were rapidly removed and placed in cold K-H buffer solution (118 mmol/L NaCl, 4.75 mmol/L KCl, 25 mmol/L NaHCO_3_, 1.18, mmol/L MgSO_4_ 1.18 mmol/L KH_2_PO_4_, 2.54 mmol/L CaCl_2_, and 11.1 mmol/L glucose), and the surrounding fat and tissue were carefully debrided. The length of the aortic rings was approximately 3 mm. The aortic segments were suspended on stainless steel hooks, aerated in K-H buffer and continuously oxygenated with 95% O_2_ at 37 °C. The aortic rings were then connected to FORT-10 force transducers for MacLab data acquisition. An initial passive tension in the aortic rings was set as 3 mN for 30 min to achieve vessel ring stability before further experimentation. The K-H buffer was replaced every 10 min. Phenylephrine was added to the tissue bath at a concentration of 10^−5 ^mol/L to induce vasoconstriction. After achieving vessel ring stability, the vasorelaxant Ach (10^−9^–10^−5 ^mol/L) was added to the tissue bath in immediate succession to obtain the dose-response curve. The relaxation at each concentration was measured and is expressed as the percentage of force generated in response to phenylephrine.

### Immunohistochemistry

Thoracic aorta tissues were fixed in formalin, dehydrated, embedded in paraffin and cut into 4 μm cross sections. These sections were incubated in citrate buffer (pH 6.0) and microwaved for 10 min twice after undergoing deparaffinization and rehydration. Goat serum (10%) (Gibco, BRL, NY, USA) was used to block non-specific binding for 1 h at 37 °C, followed by an overnight incubation with primary antibodies against p21^Cip1^ (1:1000) and p16^INK4a^ (1:200) at 4 °C in a humid box. After three washes in PBS, horseradish peroxidase (HRP)-conjugated secondary antibodies (Abcam) were added and incubated for 60 min at 37 °C, followed by diaminobenzidine (1:100, Abcam) staining. Haematoxylin was applied to counterstain the cell nuclei. The sections were covered with neutral gum and photographed under a Zeiss microscope.

### Haematoxylin-eosin (HE) staining

Paraffin‐embedded thoracic aorta sections were stained by haematoxylin for 2 min. After washing and treating with 1% acidic alcohol, the sections were treated with eosin staining for 3 min. Afterward, the sections were washed, dehydrated, treated by xylene before microscopic observation. The thoracic aorta sections were photographed under a Zeiss microscope.

### Masson’s trichrome staining

Paraffin‐embedded thoracic aorta sections were stained in Weigert’s iron haematoxylin for 10 min after undergoing deparaffinization and rehydration and was then washed with PBS for 5 min. The tissues were then stained in Biebrich scarlet-acid fuchsin solution for 5 min and washed in distilled water. The sections then underwent differentiation and dehydration in 75% and 90% alcohol a few times, followed by being rinsed in tap water. Finally, the sections were cleared in xylene and mounted with mounting medium. The sections were visualized with a microscope. Interstitial collagen deposition was indicated by blue-green staining.

### Alizarin red S staining

For aortic calcification staining, thoracic aorta sections were stained with 2% alizarin red S solution for 10 min and washed with PBS. Then, the sections were soaked in anhydrous acetone for 30 s, a mixed solution of anhydrous acetone and xylene (volume ratio = 1:1) for 15 s and anhydrous xylene for 1 min. Interstitial calcium phosphate salts were indicated by red staining.

### Western blotting

Thoracic aortas were placed in RIPA lysis buffer (HaiGene, Haerbin, China) supplemented with a protease inhibitor (MedChemExpress, Monmouth Junction, NJ, USA) and then homogenized by ultrasonication on ice. The tissue lysate was centrifuged at 12,000 × *g* at 4 °C for 15 min, and the supernatant was collected in a new tube. Afterward, the protein concentration in the supernatant was determined using a BCA protein assay kit (Beyotime Institute of Biotechnology, Jiangsu, China). SDS-PAGE was performed with equal amounts of protein from each sample as previously described (Zheng et al. 2020), and the proteins were then transferred to PVDF membranes (EMD Millipore, Billerica, MA, USA). The membranes were incubated at 4 °C overnight with one of the following primary antibodies after being blocked with 5% bovine serum albumin (Gibco) for 1 h at room temperature: p21^Cip1^ (1:1000), p16I^NK4a^ (1:1000), ICAM-1 (1:1000), VCAM-1 (1:5000), PAI-1 (1:1000), collagen I (1:1000), collagen III (1:300), Gas6 (1:1000), Axl (1:500) and GAPDH (1:1000). After being washed, the membranes were incubated with an HRP-conjugated secondary antibody (Boster, Wuhan, China) for 1 h at room temperature. Secondary antibody binding was assayed using an ECL kit (EMD Millipore), and the intensities of the bands were analysed using ImageJ software (version 1.41; National Institutes of Health, MD, USA). GAPDH expression was used as an internal control.

### Real-time quantitative RT-PCR (qPCR)

First, total RNA was isolated from thoracic aortas using a TaKaRa MiniBEST Universal RNA Extraction Kit (9767, TaKaRa) in accordance with the manufacturer’s protocols. The RNA concentration was then measured with a DS-11 FX Spectrophotometer (DeNovix). Next, the extracted RNA (1000 ng each for a 20 µL reaction system) was reverse transcribed into cDNA using PrimeScript^TM^ RT Master Mix (RR360A, TaKaRa) according to the manufacturer’s instructions in a ProFlex PCR System (Thermo Scientific). Finally, the reverse transcription products were used as templates for further PCR amplification with SYBR Premix Ex Taq II (TaKaRa). The following primer sequences were used: *Gapdh*: forward 5′-CAGCAACTCCCACTCTTCCAC-3′ and reverse 5′- TGGTCCAGGGTTTCTTACTC-3′; and *Axl*: forward 5′-GGAACCCAGGGAATATCACAGG-3′ and reverse 5′-AGTTCTAGGATCTGTCCATCTCG-3′; and *Gas6* forward 5′-TGCTGGCTTCCGAGTCTTC-3′ and reverse 5′-CGGGGTCGTTCTCGAACAC-3′. Each reaction well in a 96-well clear plate (Thermo Scientific) which contained 0.4 pmol/μL forward and reverse primers, 1× SYBR Premix Ex Taq II (RR820A, TaKaRa) and 10 ng of cDNA. The final reaction volume was 20 µL, and all samples were analysed in triplicate. cDNA amplification was performed as follows: pre-denaturation at 95 °C for 30 s, followed by 40 cycles of denaturation at 95 °C for 5 s and annealing at 60 °C for 35 s in QuantStudio5 (Thermo Scientific). The 2^–ΔΔCT^ method was used for relative quantitative analysis of the collected data. The relative mRNA expression level of target genes was obtained by comparing data from the experimental group with those of the control group with reference to *Gapdh*.

### Statistical analysis

All experiments were carried out at least three times. Statistical analyses were conducted using IBM SPSS Statistics for Windows, Version 25.0 (IBM Corp). The data are expressed as the mean ± S.D. and were analysed by one-way ANOVA. Tukey analysis was used to compare the two groups after ANOVA. *p* < 0.05 was considered statistically significant.

## Results

### Rb1 alleviated senescence in the aortas of ageing mice

**Figure 1.**
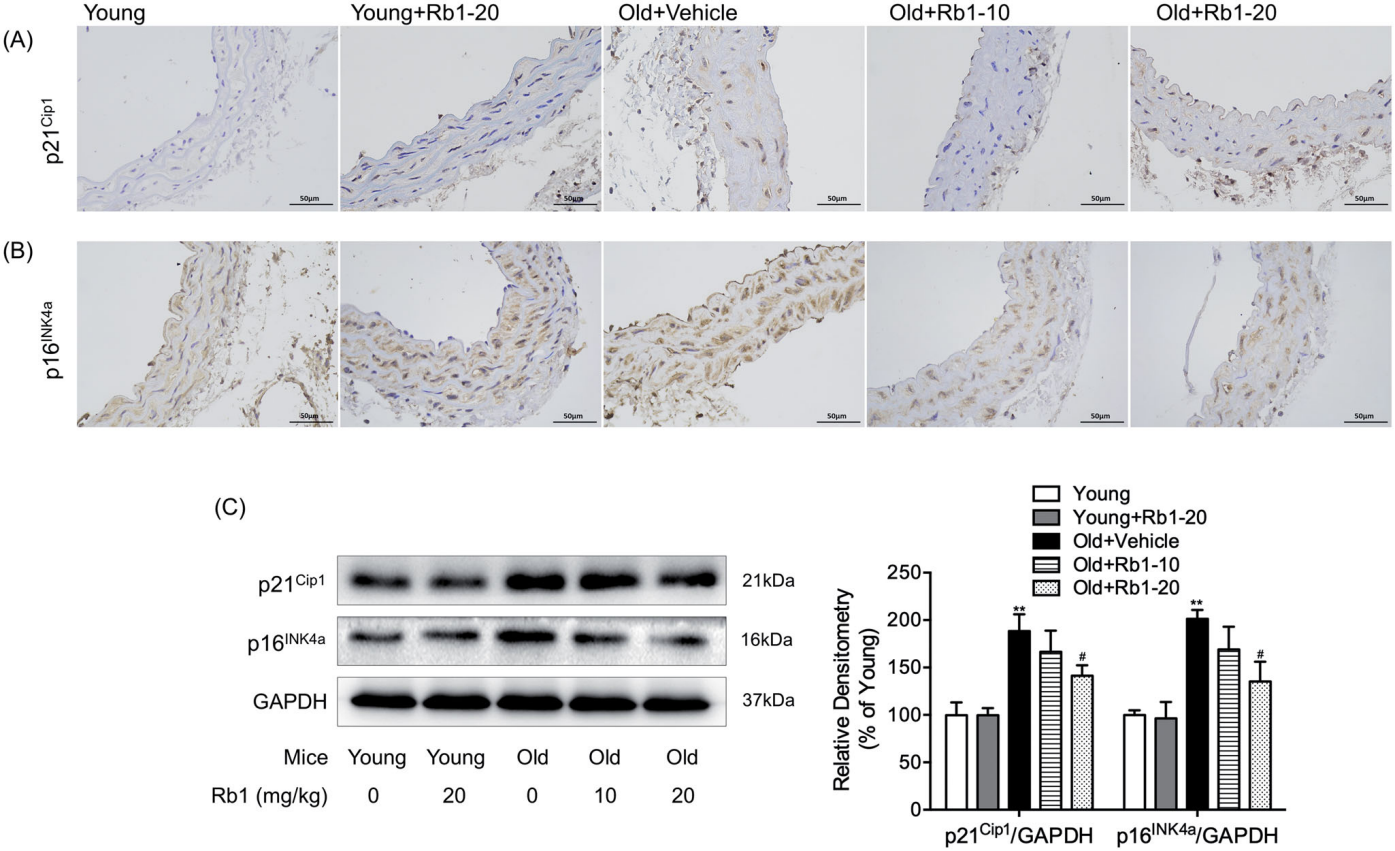
Effect of Rb1 on thoracic aorta senescence. Images of the immunohistochemical staining (400 ×) of p21^Cip1^ (A) and p16^INK4a^ (B) in mouse thoracic aorta cross sections. (C) Western blot analysis of mouse thoracic aortic p21^Cip1^ and p16^INK4a^ expression. The data are expressed as the mean ± SD. **p < 0.01 vs. the Young group; ^#^*p* < 0.05 vs. the Old + Vehicle group.

To identify the antisenescence effect of Rb1, we first investigated cellular senescence, which is characterized by the expression of proteins involved in cell cycle inhibition and irreversible growth arrest (Yang et al. 2019). As the immunohistochemistry results shown in Figure 1(A,B), compared with those in the Young group, Old + Vehicle group mice showed that ageing accelerated the upregulation of p21^Cip1^ and p16^INK4a^ in thoracic aorta cells. However, Rb1 treatment alleviated cellular senescence in blood vessels, especially in the Old + Rb1-20 group. In addition, the Western blot results were in accordance with the immunohistochemistry results. As shown in Figure 1(C), the protein expression of p21^Cip1^ and p16^INK4a^ was significantly increased by approximately 2-fold in the thoracic aortas of aged mice compared with young mice. After treatment with 20 mg/kg Rb1, the protein levels of p21^Cip1^ and p16^INK4a^ were just increased by 41.92% and 37.52% compared with those of the Young group, which were significantly lower than those of the Old + Vehicle group. In addition, there was no significant difference in the expression of p21^Cip1^ or p16^INK4a^ between young mice with and without Rb1 treatment.

### Rb1 ameliorated vascular reactivity in aged mice

**Figure 2.**
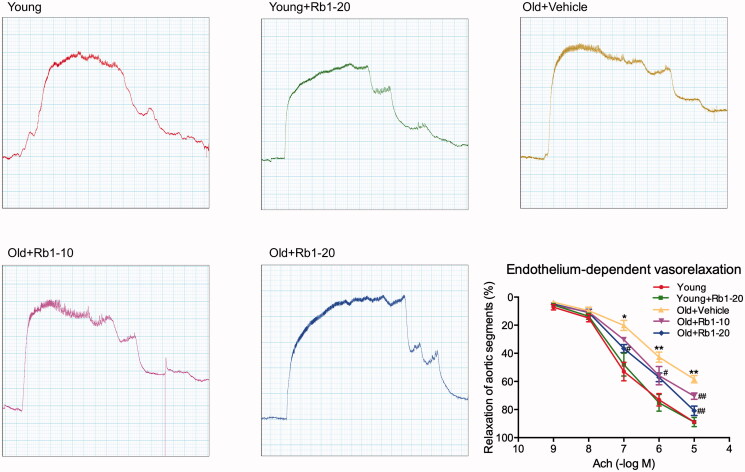
Rb1 treatment attenuated age-associated endothelium-dependent vasorelaxation impairment in thoracic aorta vascular rings. Figures showing the analysis results. The data are expressed as the mean ± SD. *p < 0.05, **p < 0.01 vs. the Young group; ^#^*p* < 0.05, ^##^*p* < 0.01 vs. the Old + Vehicle group.

A vascular ring experiment was used to evaluate endothelium-dependent vasodilatation. As shown in Figure 2, in all mouse thoracic aortic rings, the administration of Ach resulted in vasorelaxation. At maximal concentrations (10^–5 ^mol/L), Ach induced approximately 88.84% ± 1.20% and 58.35% ± 2.50% vasorelaxation in the Young group and the Old + Vehicle group, respectively, demonstrating that ageing is associated with vascular reactivity impairment. However, 10 and 20 mg/kg Rb1 treatment resulted in approximately 70.48% ± 2.20% and 80.90% ± 3.24% relaxation, respectively, in ageing mouse thoracic aortic rings in response to 10^−5 ^mol/L Ach.

### Rb1 reduced cellular adhesion molecule expression in the thoracic aortas of ageing mice

**Figure 3.**
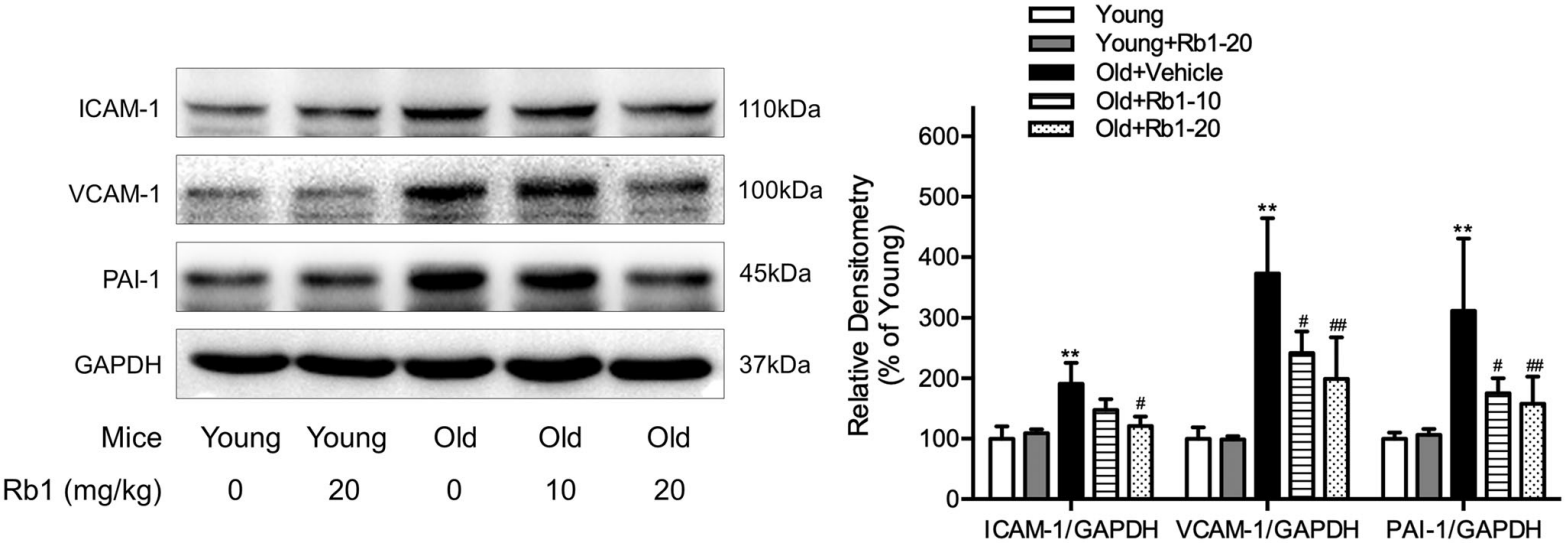
Rb1 reduced cellular adhesion molecule expression in aged mouse thoracic aortas. Western blot analysis of ICAM-1, VCAM-1 and PAI-1 expression in thoracic aorta tissues. The data are expressed as the mean ± SD. **p < 0.01 vs. the Young group; ^#^*p* < 0.05, ^##^*p* < 0.01 vs. the Old + Vehicle group.

We examined the protein expression of certain cellular adhesion molecules to determine the inflammatory and anti-inflammatory effects of Rb1 on aged mice. The results showed that the protein expression of ICAM-1, VCAM-1 and PAI-1 was significantly increased by approximately 2-4 fold in the thoracic aortas of aged mice compared with those in the Young group (Figure 3). In contrast, Rb1 treatment significantly inhibited these changes in aged mouse thoracic aortas but had no effect on the Young mouse groups. The quantitative analysis indicated that 20 mg/kg Rb1 treatment resulted in approximately 36.58%, 46.66% and 49.34% downregulation of ICAM-1, VCAM-1 and PAI-1 protein expression, respectively, in ageing thoracic aortic tissue, compared with those of Old + Vehicle group mice.

### Rb1 inhibited age-related vascular calcification and fibrosis

**Figure 4.**
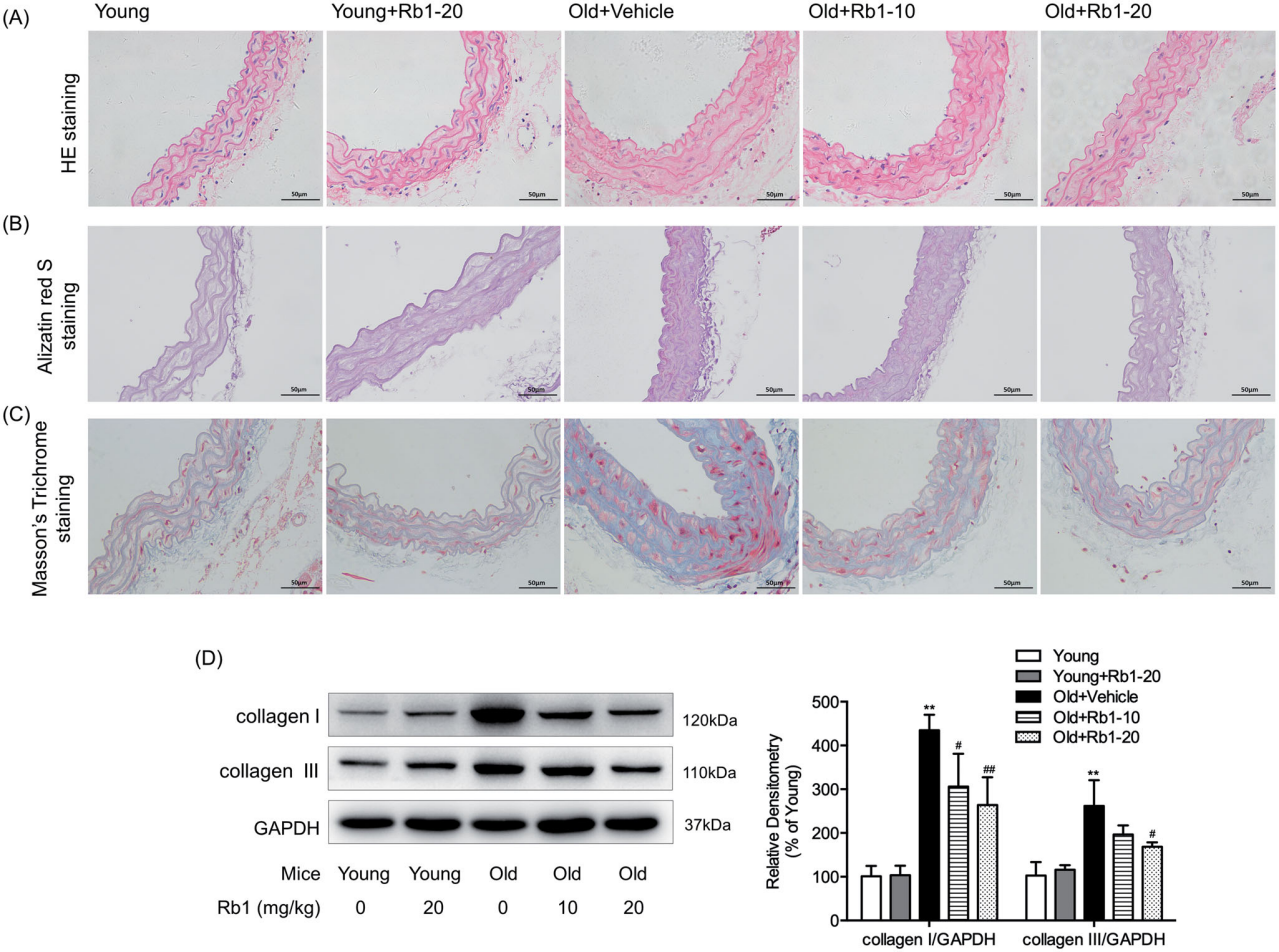
Rb1 treatment ameliorated ageing-induced vascular calcification and fibrosis. (A) Haematoxylin and eosin (HE) staining (400 ×), (B) Alizarin red S staining (400 ×) and (C) Masson’s trichrome staining (400 ×) of representative thoracic aorta sections in each group. (D) Western blot analysis of collagen I and collagen III expression in thoracic aorta tissues. The data are expressed as the mean ± SD. **p < 0.01 vs. the Young group; ^#^*p* < 0.05, ^##^*p* < 0.01 vs. the Old + Vehicle group.

We performed HE staining and alizarin red S staining to assess aortic tissue structure and vascular medial calcification in ageing mice respectively. HE staining showed that ageing induced thickened wall of thoracic aorta and disorganisation of the aortic extracellular matrix, which could be relieved by Rb1 treatment (Figure 4(A)). As shown in Figure 4(B), ageing mice developed more severe medial calcification (red-stained area) than young mice, while Rb1 intervention reduced this pathological change. Then, we examined thoracic aorta cross sections by Masson’s trichrome staining to assess vascular medial fibrosis in ageing mice. The results showed that there was a noticeable increase in collagen deposition (blue-stained area) in the Old + Vehicle group compared with the Young group (Figure 4(C)), and this effect was significantly inhibited by Rb1 intervention. Moreover, Western blotting showed that the expression of collagen I and collagen III significantly increased by 4.34- and 2.62-fold in Old + Vehicle group mice compared with young mice; however, Rb1 treatment downregulated the expression of collagen I and collagen III in aged mice (Figure 4(D)). Moreover, treatment with Rb1 had no influence on the degrees of thoracic aorta calcification and fibrosis in young mice (Figure 4(A–D)).

### Rb1 regulated the Gas6 signalling pathway

**Figure 5.**
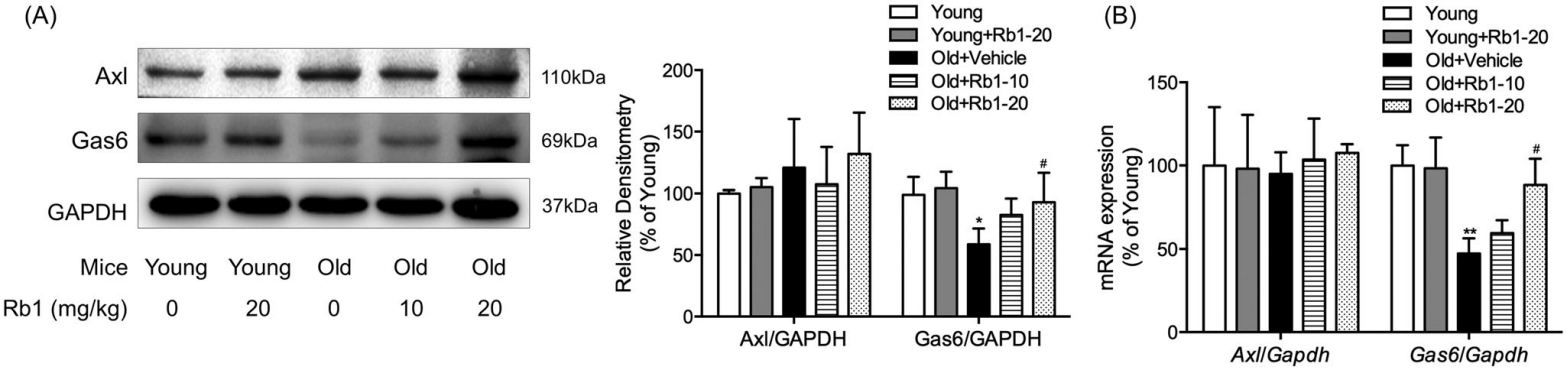
The influence of Rb1 on the Gas6/Axl signalling pathway. (A) Western blotting was performed to examine the protein expression of Gas6 and Axl. (B) qPCR was performed to examine the mRNA expression of *Gas6* and *Axl*. The data are expressed as the mean ± SD. *p < 0.05, **p < 0.01 vs. the Young group; ^#^*p* < 0.05 vs. the Old + Vehicle group.

We further investigated whether Rb1 functioned through the Gas6/Axl signalling pathway to ameliorate age-associated thoracic aorta impairment in mice. The downregulation of Gas6 protein expression by 41.72% and mRNA expression by 52.73% in aged mice compared with young mice was abrogated by Rb1 treatment (Figure 5(A,B)). We also determined the protein expression of Axl by Western blotting. Interestingly, there was no significant difference in Axl protein expression among the five experimental groups (Figure 5(A)). We also did not detect a significant difference in the mRNA levels of *Axl* among the groups (Figure 5(B)).

## Discussion

In the present study, we demonstrated that Rb1 ameliorated age-related vascular impairment by suppressing calcification and fibrosis through the regulation of Gas6 expression but not Axl expression, which provides a potential therapeutic strategy for preventing age-related vascular disease.

It is well accepted that vascular cell senescence, which is characterized by endothelial dysfunction and the phenotypic transition of smooth muscle cells, can result in increased vascular stiffness and increased thickness of vascular walls (Liu et al. 2019). Vascular cell senescence is a common feature of various complex, age-related diseases, particularly cardiovascular diseases (O'Rourke et al. 2010). With the development of vascular cell senescence, vascular function is impaired (Gong et al. 2014). Age-related vascular dysfunction is exemplified by pathophysiological alterations, including a progressive decline in endothelium-dependent vasodilatation (Herrera et al. 2010; Gong et al. 2014). In our study, we confirmed cellular senescence in the thoracic aortas of aged mice, which was indicated by increased expression of the age-related proteins p21^Cip1^ and p16^INK4a^, as well as a decline in endothelium-dependent vasodilatation. The consistency of our results with the above-mentioned studies suggests that there were some important molecular mechanisms associated with the changes in vascular cell pathophysiology during ageing. Agents that act on these mechanisms would probably greatly reduce and delay vascular cell senescence.

Accumulating evidence has revealed that Rb1 is considered a promising drug for preventing vascular damage (Zhou et al. 2017, 2019a, 2019b; Zheng et al. 2020). Previous studies have revealed that Rb1 prevents vascular cell senescence and inhibits vascular calcification (Nanao-Hamai et al. 2019; Zhou et al. 2019b; Zheng et al. 2020). However, it is still uncertain whether Rb1 can protect arterial functions against ageing and the relevant molecular mechanism. Here, we found that Rb1 reduced and delayed arterial senescence in aged mice, as demonstrated by decreased expression of related proteins and improved vascular vasodilatation, indicating that Rb1 can efficiently block excessive senescence in ageing mouse thoracic aortas and might be used as an effective agent to treat or prevent age-related arterial impairment.

Previous evidence has demonstrated the accumulation of chronic low-grade arterial inflammation with advancing age, which contributes to age-associated vascular structural and functional alterations (Wang et al. 2014). Additional studies have indicated that a microenvironment enriched in inflammatory profiles induces a phenotypic shift in VSMCs from the contractile to the synthetic type. This phenotypic shift is characterized by the attenuated expression of SMC-specific contractile proteins and the secretion of additional proinflammatory cytokines, chemokines, ECM proteins, and cell adhesion molecules, such as collagen, ICAM-1, VCAM-1 and PAI-1 (Wang et al. 2011; Lacolley et al. 2018). Moreover, adhesion molecules contribute to inflammation-induced remodelling of the arteries, particularly by reorganising ECM-VSMC interactions and influencing the phenotypic modulation of VSMCs (Intengan et al. 1999). Furthermore, studies have shown that Rb1 has anti-inflammatory effects *in vivo* and *in vitro* (Miao et al. 2017; Zhou et al. 2017). In the present study, we found that Rb1 treatment could significantly decrease the expression levels of cell adhesion molecules related to inflammatory processes in aged mice, suggesting that Rb1 might act as an anti-inflammatory drug and protect the vasculature during ageing.

In addition to inflammatory damage, remodelling of the arterial wall characterized by calcification and fibrosis is the other main pathophysiological change in age-related avascular modifications and causes vascular stiffness that contributes to a decline in vasodilatation (McEniery et al. 2005; Iyemere et al. 2006; Kovacic et al. 2011). Large-scale studies have demonstrated that coronary atherosclerotic calcification increases with age (Tesauro et al. 2017). Other research also demonstrated the presence of increased calcification with ageing in the carotid artery region (Wendorff et al. 2015). Similarly, our results indicated that ageing mice developed more severe vascular medial calcification than young mice. Rb1 intervention substantially inhibited these changes. This result suggested that Rb1 administration alleviated the degree of age-related vascular calcification *in vivo*. Sufficient evidence has shown that vascular calcification involves a variety of pathobiological processes rather than simple calcium deposition, among which VSMC switching into osteoblast-like cells play a critical role (Zhou et al. 2019b). One limitation of our study is that, despite revealing that Rb1 intervention significantly inhibited vascular calcification in ageing mice thoracic aortas, contractile VSMC markers (such as A‐smooth muscle actin and calponin) and osteogenic VSMC markers (such as runt‐related transcription factor 2) require further exploration.

Moreover, in the present study, we also observed decreased interstitial collagen levels and decreased related-protein expression in Rb1-treated aged mice compared with vehicle-treated aged mice, which suggested that Rb1 might have a beneficial effect on vascular function in ageing thoracic aortas by ameliorating age-induced vascular fibrosis. Harvey et al. (2016) reported that arterial stiffening caused by excessive fibrosis and increased collagen deposition could result in increased vasomotor tone and altered tissue perfusion. Agents that attenuate fibrosis are considered to protect against age-related vascular diseases (Kim et al. 2018).

It can be inferred that age-related vascular impairment is a complex process that involves various abnormal changes, including inflammation, calcification and fibrosis. More importantly, we found that Rb1 was able to inhibit these known abnormal changes in the thoracic aortas of aged mice. However, the underlying mechanism is still unknown. Gas6 was identified as the ligand for the TAM receptor family, especially Axl tyrosine kinase receptors (Rothlin et al. 2015). A previous study demonstrated the roles of Gas6/Axl in regulating multiple cellular functions (Chen et al. 2019). A recent study suggested that Gas6/Axl played a crucial role in vascular calcification (Son et al. 2010; Kaesler et al. 2016; Nanao-Hamai et al. 2019) and may also be involved in treatment-induced downregulation of collagen synthesis in VSMCs and ageing heart tissue (Chen et al. 2016; 2019). Moreover, previous studies revealed that Rb1 activated Gas6 transcription at the specific ARE site in the promoter region (Son et al. 2010; Nanao-Hamai et al. 2019). Here, we demonstrated that Gas6 protein expression was downregulated in ageing mice but was reversed by Rb1 treatment, indicating that Gas6 may participate in Rb1-mediated regulation of arterial impairment in aged mice. However, our results show that there was no change in Axl protein and mRNA expression, which was inconsistent with a previous study (Chen et al. 2019). The TAM receptor family includes Tyro3, Axl and MerTK. Although Gas6 shows the strongest affinity for Axl among the three members (Rothlin et al. 2015), Sun et al. (2019) found that Gas6 activation attenuated inflammatory injury and apoptosis in mice, but no changes in Axl activation occurred among the experimental groups. Other research suggested that Axl expression, which inhibits apoptosis and thus vascular calcification, was also unaltered in Gas6–/– mice (Kaesler et al. 2016). Our results clearly indicated that Rb1 specifically attenuated age-related Gas6 downregulation but had no influence on the expression of Axl, which was reported to be the primary event responsible for vascular calcification and fibrosis, to improve age-related vascular inflammation and endothelium-dependent vasodilatation. The stable expression of Axl may result from a sufficient quantity before Gas6 activation or indicate that Gas6 functions through other receptors. Further investigation exploring the effects of Rb1 on other TAMs that could also be activated by Gas6, such as Tyro3 and MerTK, is needed.

## Conclusions

In the present study, we confirmed that Rb1 inhibited vascular calcification and fibrosis and decreased the expression of cellular adhesion molecules, all of which subsequently ameliorated age-related vascular endothelium-dependent vasodilatation at least in part by regulating Gas6 expression but not Axl expression. With uncontrollable ageing, finding agents that can prevent or treat age-related vascular cell impairment is of great importance. Our study provides evidence validating the effects of Rb1 on ameliorating vascular injury in ageing mice.

## Data Availability

Data and materials are available upon request to the corresponding author.

## References

[CIT0001] Acosta JC, O’Loghlen A, Banito A, Guijarro MV, Augert A, Raguz S, Fumagalli M, Da Costa M, Brown C, Popov N, et al. 2008. Chemokine signaling via the CXCR2 receptor reinforces senescence. Cell. 133(6):1006–1018. 18555777 10.1016/j.cell.2008.03.038

[CIT0002] Brandes RP, Fleming I, Busse R. 2005. Endothelial aging. Cardiovasc Res. 66(2):286–294. 15820197 10.1016/j.cardiores.2004.12.027

[CIT0003] Chen FF, Song FQ, Chen YQ, Wang ZH, Li YH, Liu MH, Li Y, Song M, Zhang W, Zhao J, et al. 2019. Exogenous testosterone alleviates cardiac fibrosis and apoptosis via Gas6/Axl pathway in the senescent mice. Exp Gerontol. 119:128–137. 30710682 10.1016/j.exger.2019.01.029

[CIT0004] Chen YQ, Zhao J, Jin CW, Li YH, Tang MX, Wang ZH, Zhang W, Zhang Y, Li L, Zhong M. 2016. Testosterone delays vascular smooth muscle cell senescence and inhibits collagen synthesis via the Gas6/Axl signaling pathway. Age. 38(3):60. 27206970 10.1007/s11357-016-9910-5 PMC5005950

[CIT0005] Fridell YW, Villa J, Attar EC, Liu ET. 1998. GAS6 induces Axl-mediated chemotaxis of vascular smooth muscle cells. J Biol Chem. 273(12):7123–7126. 9507025 10.1074/jbc.273.12.7123

[CIT0006] Gong X, Ma Y, Ruan Y, Fu G, Wu S. 2014. Long-term atorvastatin improves age-related endothelial dysfunction by ameliorating oxidative stress and normalizing eNOS/iNOS imbalance in rat aorta. Exp Gerontol. 52:9–17. 24463049 10.1016/j.exger.2014.01.015

[CIT0007] Harvey A, Montezano AC, Lopes RA, Rios F, Touyz RM. 2016. Vascular fibrosis in aging and hypertension: molecular mechanisms and clinical implications. Can J Cardiol. 32(5):659–668. 27118293 10.1016/j.cjca.2016.02.070 PMC4906153

[CIT0008] Herrera MD, Mingorance C, Rodriguez-Rodriguez R, Alvarez de Sotomayor M. 2010. Endothelial dysfunction and aging: an update. Ageing Res Rev. 9(2):142–152. 19619671 10.1016/j.arr.2009.07.002

[CIT0009] Hsu JJ, Lu J, Umar S, Lee JT, Kulkarni RP, Ding Y, Chang CC, Hsiai TK, Hokugo A, Gkouveris I, Tetradis S, et al. 2018. Effects of teriparatide on morphology of aortic calcification in aged hyperlipidemic mice. Am J Physiol Heart Circ Physiol. 314(6):H1203–H1213. 29451816 10.1152/ajpheart.00718.2017 PMC6032086

[CIT0010] Hurtado B, Abasolo N, Munoz X, Garcia N, Benavente Y, Rubio F, Garcia de Frutos P, Krupinski J, Sala N. 2010. Association study between polymorphims in GAS6-TAM genes and carotid atherosclerosis. Thromb Haemost. 104(3):592–598. 20664904 10.1160/TH09-11-0787

[CIT0011] Intengan HD, Thibault G, Li JS, Schiffrin EL. 1999. Resistance artery mechanics, structure, and extracellular components in spontaneously hypertensive rats: effects of angiotensin receptor antagonism and converting enzyme inhibition. Circulation. 100(22):2267–2275. 10578002 10.1161/01.cir.100.22.2267

[CIT0012] Iyemere VP, Proudfoot D, Weissberg PL, Shanahan CM. 2006. Vascular smooth muscle cell phenotypic plasticity and the regulation of vascular calcification. J Intern Med. 260(3):192–210. 16918817 10.1111/j.1365-2796.2006.01692.x

[CIT0013] Kaesler N, Immendorf S, Ouyang C, Herfs M, Drummen N, Carmeliet P, Vermeer C, Floege J, Kruger T, Schlieper G. 2016. Gas6 protein: its role in cardiovascular calcification. BMC Nephrol. 17(1):52. 27230889 10.1186/s12882-016-0265-z PMC4880820

[CIT0014] Kim EN, Kim MY, Lim JH, Kim Y, Shin SJ, Park CW, Kim YS, Chang YS, Yoon HE, Choi BS. 2018. The protective effect of resveratrol on vascular aging by modulation of the renin-angiotensin system. Atherosclerosis. 270:123–131. 29407880 10.1016/j.atherosclerosis.2018.01.043

[CIT0015] Korshunov VA, Mohan AM, Georger MA, Berk BC. 2006. Axl, a receptor tyrosine kinase, mediates flow-induced vascular remodeling. Circ Res. 98(11):1446–1452. 16627783 10.1161/01.RES.0000223322.16149.9a

[CIT0016] Kovacic JC, Moreno P, Hachinski V, Nabel EG, Fuster V. 2011. Cellular senescence, vascular disease, and aging: Part 1 of a 2-part review. Circulation. 123(15):1650–1660. 21502583 10.1161/CIRCULATIONAHA.110.007021

[CIT0017] Kwok HH, Yue PY, Mak NK, Wong RN. 2012. Ginsenoside Rb1 induces type I collagen expression through peroxisome proliferator-activated receptor-delta. Biochem Pharmacol. 84(4):532–539. 22692056 10.1016/j.bcp.2012.05.023

[CIT0018] Lacolley P, Regnault V, Avolio AP. 2018. Smooth muscle cell and arterial aging: basic and clinical aspects. Cardiovasc Res. 114(4):513–528. 29514201 10.1093/cvr/cvy009

[CIT0019] Lakatta EG, Levy D. 2003. Arterial and cardiac aging: major shareholders in cardiovascular disease enterprises: Part I: aging arteries: a “set up” for vascular disease. Circulation. 107(1):139–146. 12515756 10.1161/01.cir.0000048892.83521.58

[CIT0020] Liu H, Wang H, Yang S, Qian D. 2019. Downregulation of miR-542-3p promotes osteogenic transition of vascular smooth muscle cells in the aging rat by targeting BMP7. Hum Genomics. 13(1):67. 31829291 10.1186/s40246-019-0245-z PMC6907335

[CIT0021] Mauriello A, Orlandi A, Palmieri G, Spagnoli LG, Oberholzer M, Christen H. 1992. Age-related modification of average volume and anisotropy of vascular smooth muscle cells. Pathol Res Pract. 188(4–5):630–636. 1409102 10.1016/S0344-0338(11)80070-1

[CIT0022] McClelland RL, Chung H, Detrano R, Post W, Kronmal RA. 2006. Distribution of coronary artery calcium by race, gender, and age: results from the Multi-Ethnic Study of Atherosclerosis (MESA). Circulation. 113(1):30–37. 16365194 10.1161/CIRCULATIONAHA.105.580696

[CIT0023] McEniery CM, Yasmin Hall IR, Qasem A, Wilkinson IB, Cockcroft JR, Investigators A. 2005. Normal vascular aging: differential effects on wave reflection and aortic pulse wave velocity: the Anglo-Cardiff Collaborative Trial (ACCT). J Am Coll Cardiol. 46:1753–1760. 16256881 10.1016/j.jacc.2005.07.037

[CIT0024] Miao HH, Zhang Y, Ding GN, Hong FX, Dong P, Tian M. 2017. Ginsenoside Rb1 attenuates isoflurane/surgery-induced cognitive dysfunction via inhibiting neuroinflammation and oxidative stress. Biomed Environ Sci. 30(5):363–372. 28549492 10.3967/bes2017.047

[CIT0025] Minamino T, Komuro I. 2007. Vascular cell senescence: contribution to atherosclerosis. Circ Res. 100(1):15–26. 17204661 10.1161/01.RES.0000256837.40544.4a

[CIT0026] Moslehi J, DePinho RA, Sahin E. 2012. Telomeres and mitochondria in the aging heart. Circ Res. 110(9):1226–1237. 22539756 10.1161/CIRCRESAHA.111.246868 PMC3718635

[CIT0027] Nakano T, Ishimoto Y, Kishino J, Umeda M, Inoue K, Nagata K, Ohashi K, Mizuno K, Arita H. 1997. Cell adhesion to phosphatidylserine mediated by a product of growth arrest-specific gene 6. J Biol Chem. 272(47):29411–29414. 9367994 10.1074/jbc.272.47.29411

[CIT0028] Nakano-Kurimoto R, Ikeda K, Uraoka M, Nakagawa Y, Yutaka K, Koide M, Takahashi T, Matoba S, Yamada H, Okigaki M, et al. 2009. Replicative senescence of vascular smooth muscle cells enhances the calcification through initiating the osteoblastic transition. Am J Physiol Heart Circ Physiol. 297(5):H1673–1684. 19749165 10.1152/ajpheart.00455.2009

[CIT0029] Nanao-Hamai M, Son BK, Komuro A, Asari Y, Hashizume T, Takayama KI, Ogawa S, Akishita M. 2019. Ginsenoside Rb1 inhibits vascular calcification as a selective androgen receptor modulator. Eur J Pharmacol. 859:172546. 31319068 10.1016/j.ejphar.2019.172546

[CIT0030] O’Rourke MF, Safar ME, Dzau V. 2010. The Cardiovascular Continuum extended: aging effects on the aorta and microvasculature. Vasc Med. 15(6):461–468. 21056945 10.1177/1358863X10382946

[CIT0031] Qin Q, Mehta H, Yen K, Navarrete G, Brandhorst S, Wan J, Delrio S, Zhang X, Lerman LO, Cohen P, et al. 2018. Chronic treatment with the mitochondrial peptide humanin prevents age-related myocardial fibrosis in mice. Am J Physiol Heart Circ Physiol. 315(5):H1127–H1136. 30004252 10.1152/ajpheart.00685.2017 PMC6415743

[CIT0032] Rothlin CV, Carrera-Silva EA, Bosurgi L, Ghosh S. 2015. TAM receptor signaling in immune homeostasis. Annu Rev Immunol. 33:355–391. 25594431 10.1146/annurev-immunol-032414-112103 PMC4491918

[CIT0033] Son BK, Akishita M, Iijima K, Ogawa S, Maemura K, Yu J, Takeyama K, Kato S, Eto M, Ouchi Y. 2010. Androgen receptor-dependent transactivation of growth arrest-specific gene 6 mediates inhibitory effects of testosterone on vascular calcification. J Biol Chem. 285(10):7537–7544. 20048160 10.1074/jbc.M109.055087 PMC2844201

[CIT0034] Sun X, Guan H, Peng S, Zhao Y, Zhang L, Wang X, Li C, Shan Z, Teng W. 2019. Growth arrest-specific protein 6 (Gas6) attenuates inflammatory injury and apoptosis in iodine-induced NOD.H-2h4 mice. Int Immunopharmacol. 73:333–342. 31129420 10.1016/j.intimp.2019.04.038

[CIT0035] Tesauro M, Mauriello A, Rovella V, Annicchiarico-Petruzzelli M, Cardillo C, Melino G, Di Daniele N. 2017. Arterial ageing: from endothelial dysfunction to vascular calcification. J Intern Med. 281(5):471–482. 28345303 10.1111/joim.12605

[CIT0036] Tolle M, Reshetnik A, Schuchardt M, Hohne M, van der Giet M. 2015. Arteriosclerosis and vascular calcification: causes, clinical assessment and therapy. Eur J Clin Invest. 45(9):976–985. 26153098 10.1111/eci.12493

[CIT0037] Wang M, Jiang L, Monticone RE, Lakatta EG. 2014. Proinflammation: the key to arterial aging. Trends Endocrinol Metab. 25(2):72–79. 24365513 10.1016/j.tem.2013.10.002 PMC3917314

[CIT0038] Wang M, Spinetti G, Monticone RE, Zhang J, Wu J, Jiang L, Khazan B, Telljohann R, Lakatta EG. 2011. A local proinflammatory signalling loop facilitates adverse age-associated arterial remodeling. PLoS One. 6(2):e16653. 21347430 10.1371/journal.pone.0016653 PMC3035650

[CIT0039] Wendorff C, Wendorff H, Pelisek J, Tsantilas P, Zimmermann A, Zernecke A, Kuehnl A, Eckstein HH. 2015. Carotid plaque morphology is significantly associated with sex, age, and history of neurological symptoms. Stroke. 46(11):3213–3219. 26451032 10.1161/STROKEAHA.115.010558

[CIT0040] Yanagita M, Arai H, Ishii K, Nakano T, Ohashi K, Mizuno K, Varnum B, Fukatsu A, Doi T, Kita T. 2001. Gas6 regulates mesangial cell proliferation through Axl in experimental glomerulonephritis. Am J Pathol. 158(4):1423–1432. 11290560 10.1016/S0002-9440(10)64093-X PMC1891897

[CIT0041] Yang D, Xiao C, Long F, Wu W, Huang M, Qu L, Liu X, Zhu Y. 2019. Fra-1 plays a critical role in angiotensin II-induced vascular senescence. FASEB J. 33(6):7603–7614. 30892941 10.1096/fj.201801671RRRR

[CIT0042] Zheng X, Wang S, Zou X, Jing Y, Yang R, Li S, Wang F. 2017. Ginsenoside Rb1 improves cardiac function and remodeling in heart failure. Exp Anim. 66(3):217–228. 28367863 10.1538/expanim.16-0121 PMC5543242

[CIT0043] Zheng Z, Wang M, Cheng C, Liu D, Wu L, Zhu J, Qian X. 2020. Ginsenoside Rb1 reduces H_2_O_2_-induced HUVEC dysfunction by stimulating the sirtuin-1/AMP-activated protein kinase pathway. Mol Med Rep. 22(1):247–256. 32377712 10.3892/mmr.2020.11096 PMC7248484

[CIT0044] Zhou J, Walker A. 2021. The impact of community care services on the preference for ageing in place in urban China. Health Soc Care Community. 29(4):1041–1050. 32783285 10.1111/hsc.13138

[CIT0045] Zhou P, Lu S, Luo Y, Wang S, Yang K, Zhai Y, Sun G, Sun X. 2017. Attenuation of TNF-α-induced inflammatory injury in endothelial cells by ginsenoside Rb1 via inhibiting NF-κB, JNK and p38 signaling pathways. Front Pharmacol. 8:464. 28824425 10.3389/fphar.2017.00464 PMC5540891

[CIT0046] Zhou P, Xie WJ, He SB, Sun YF, Meng XB, Sun GB. 2019a. Ginsenoside Rb1 as an anti-diabetic agent and its underlying mechanism analysis. Cells. 8:204. 10.3390/cells8030204 PMC6468558 30823412

[CIT0047] Zhou P, Zhang X, Guo M, Guo R, Wang L, Zhang Z, Lin Z, Dong M, Dai H, Ji X, et al. 2019b. Ginsenoside Rb1 ameliorates CKD-associated vascular calcification by inhibiting the Wnt/β-catenin pathway. J Cell Mol Med. 23(10):7088–7098. 31423730 10.1111/jcmm.14611 PMC6787443

